# IFN-γ promoted exosomes from mesenchymal stem cells to attenuate colitis via miR-125a and miR-125b

**DOI:** 10.1038/s41419-020-02788-0

**Published:** 2020-07-30

**Authors:** Ruili Yang, Huaming Huang, Shengjie Cui, Yikun Zhou, Ting Zhang, Yanheng Zhou

**Affiliations:** 1 Department of Orthodontics, Peking University School and Hospital of Stomatology, Haidian District, Beijing, China; 2 National Clinical Research Center for Oral Diseases & National Engineering Laboratory for Digital and Material Technology of Stomatology, Haidian District, Beijing, China; 3 Beijing Key Laboratory of Digital Stomatology, Haidian District, Beijing, China

**Keywords:** Mechanisms of disease, Stem-cell research

## Abstract

Bone marrow mesenchymal stem cells (MSCs) have demonstrated therapeutic effects for colitis through immunomodulation and anti-inflammation. However, whether MSC-derived exosomes possessed the similar function remains unclear. In present study, exosomes were isolated from control and IFN-γ-primed MSCs and was verified by transmission electron microscope (TEM) and immunofluorescence staining. Administration of exosomes to mice significantly improved the disease activity index and histological score of colitis, and decreased the ratio of Th17 cells with elevated Treg cells ratio in mice colitis model. Exosomes from IFN-γ-primed MSCs showed superior therapeutic effects to colitis. Exosomes treatment inhibited Th17 differentiation in vitro, and exosomes from IFN-γ-primed MSCs showed higher inhibition efficacy. Mechanistically, exosomes treatment significantly decreased the expression of Stat3 and p-Stat3 to inhibit Th17 cells differentiation. IFN-γ pretreatment increased the level of miR-125a and miR-125b of exosomes, which directly targeted on Stat3, to repress Th17 cell differentiation. Moreover, combination of miR-125a and miR-125b agmior infusion also showed therapeutic effects for colitis, accompanied by decreased Th17 cell ratio. Collectively, this study demonstrates that IFN-γ treatment promoted exosomes from MSCs to attenuate colitis through increasing the level of miR-125a and miR-125b, which binding on 3′-UTR of Stat3 to repress Th17 cell differentiation. This study provides a new approach of exocytosis on the treatment of colitis.

## Introduction

Mesenchymal stem cells (MSCs) have displayed tremendous therapeutic effects for variety of diseases due to their multilineage differentiation and strong immunomodulatory ability^1–4^. The immunomodulatory property of MSCs was reported that largely attributed to their secreted paracrine effectors, which exerts therapeutic effect instead of MSC itself to replace damaged tissues directly. Studies have showed that MSCs regulated immune cells via secretion of soluble molecules, such as nitric oxide, transforming growth factor (TGF)-β, prostaglandin E2 (PGE2), indoleamine 2,3-dioxygenase (IDO), galectins, and programmed death ligand^1,2,5–9^. Despite numerous molecules secreted by MSCs showed immunosuppressive properties, no specific mediators have been identified to be responsible for the therapeutic effects of MSCs. Among the paracrine effectors, extracellular vesicle particular exosomes secreted by MSCs have drawn more attentions recently due to their abundant components and anti-inflammatory function.

Exosomes, 30–150-nm lipid bilayer membrane vesicles, could be isolated from variety of cells including MSCs and released into the extracellular medium^10,11^. Administration of exosomes derived from MSCs has been reported to ameliorate several disease phenotypes in animal model. For example, exosomes from human adipose tissue-derived MSCs were reported to reduce the levels of lgE, infiltrated mast cells, and eosinophils in mouse atopic dermatitis^12^. MSC-derived exosomes could also attenuate the symptoms of experimental autoimmune encephalomyelitis (EAE) via reducing proinflammatory cytokines and promoting regulatory T (Treg) cells^13^. Recently, several researches have also showed that exosomes from MSCs reduced murine inflammatory bowel disease (IBD)^14–16^ because the characteristics of exosomes vary depending on the status of the MSCs, which alters in response to external stimuli^17^. Moreover, studies have showed that cytokines licensed MSCs are more effective to treat some diseases such as EAE and colitis via promoting the immunomodulation of MSCs after IFN-γ and TNF-α treatment^18–20^. Whether IFN-γ treatment could increase the therapeutic potential of MSC-derived exosomes and whether it could regulate the property of exosomes directly or indirectly? All these issues remain elusive.

Therefore, we isolated exosomes from control and IFN-γ-primed MSCs to treat dextran sulfate sodium (DSS)-induced colitis model to explore their therapeutic effects and the underlying mechanisms. The results demonstrated that IFN-γ treatment could enhance the capacity of exosomes derived from MSCs to attenuate mice colitis phenotype via upregulating the level of miR-125a and miR-125b, which directly targeting on the 3′-UTR of Stat3 to repress Th17 cells and the inflammation.

## Results

### The effects of IFN-γ on MSC-derived exosomes

**Fig. 1:**
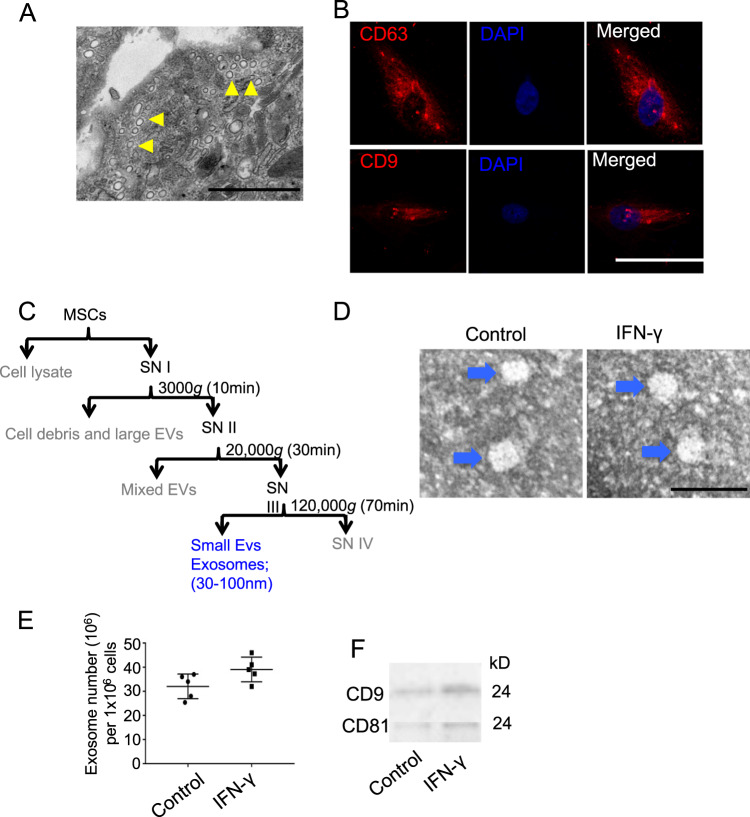
Characteristic of exosomes isolated from mouse BMMSCs. **a** Microvesicles in MSCs were analyzed by TEM. **b** Immunofluorescent staining of CD63 and CD9 in MSCs. **c** Differential centrifugation procedure for the isolation of exosomes from culture supernatants of MSCs (SN). **d** Isolated exosomes from control and IFN-γ-primed MSCs were analyzed by TEM. **e** Exosomes numbers isolated from control and IFN-γ-primed MSCs was analyzed by EXOCEP exosome quantitation kit. **f** The expression of CD81 and CD9 in exosomes from control and IFN-γ-primed MSCs were analyzed by western blotting. Scale bar **a**: 500 nm, **b**: 20 μm, and **d**: 100 nm. *P < 0.05, **P < 0.01, ***P < 0.001.

To isolate and analyze the exosomes derived from MSCs, we first analyzed mouse bone marrow MSCs using TEM and found that microvesicles were accumulated in the cytoplasm of MSCs (Fig. 1a). Then, we did immunofluorescence staining using the exosomes markers CD63 and CD9, and the results showed that CD63 and CD9 were expressed in MSCs (Fig. 1b). Then, exosomes from control and 50 ng/ml IFN-γ-primed MSCs were isolated using ultracentrifuge strategy (Figs. 1c and S1) and identified the purity using TEM that showed that the control and IFN-γ-primed-MSC exosomes were predominantly round and ranged in size from 30 to 150 nm (Fig. 1d). IFN-γ treatment slightly increases the number of exosomes secreted by MSCs (Fig. 1e); however, it increased the expression of CD9 and CD81 in exosomes significantly, as assessed by western blot (Fig. 1f).

### IFN-γ pretreatment promoted the therapeutic effects of exosomes to treat colitis

**Fig. 2:**
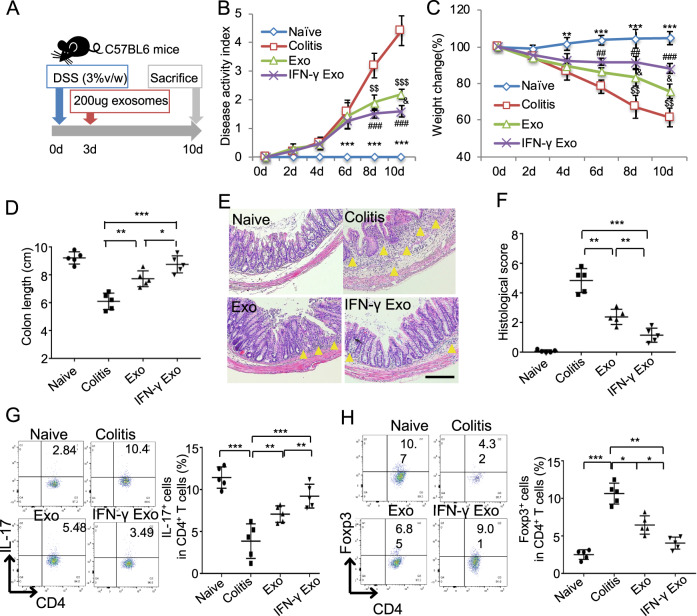
IFN-γ primes could promote exosomes to attenuate colitis in mice. **a** The schematic of exosomes infusion in DSS-induced colitis in mice. **b**, **c** The disease activity index (**b**) and mice body weight change (**c**) in control, DSS, and control MSC exosomes and IFN-γ-primed-MSC exosomes infusion group (*n* = 5). **d** The colon length in control, DSS, and control MSC exosomes and IFN-γ-primed-MSC exosomes infusion group. **e**, **f** IFN-γ-primed-MSC exosomes infusion showed superior effects to reduce inflammation cells infiltration in colon, as assessed by HE staining (**e**) and reduce histological score caused by DSS administration, compared with control exosomes infusion group (**f**). **g**, **h** Th17 cells (**g**) and Treg cells (**h**) in control, DSS, control MSC exosomes and IFN-γ-primed-MSC exosomes infusion group, assessed by flow cytometry. Scale bar: 100 μm (*n* = 5) d days, DSS dextran sulfide sodium, Exo exosomes, IFN-γ Exo exosomes derived from IFN-γ-primed MSC. The yellow triangle: inflammatory cells infiltration. *P < 0.05, **P < 0.01, ***P < 0.001; ^#^IFN-γ Exo vs colitis; ^&^IFN-γ Exo vs Exo; ^$^Exo vs colitis; *naive vs colitis.

To compare the therapeutic effects of exosomes from control and IFN-γ-primed MSCs, we induced experimental colitis by administrating 3% DSS in drink water for 10 days using C57BL/6J mice. Two hundred micrograms of exosomes derived from control or IFN-γ-primed MSCs were systemically infused to mice on day 3 and sacrifice the mice on day 10 for further evaluations (Fig. 2a). Consistent with the previous report^21^, DSS-induced colitis mice appeared a sustained body weight loss and presence of loose feces/bloody diarrhea, which were evaluated as increased disease activity index (DAI). Infusion of control MSC exosomes retarded body weight loss of colitis mice, as well as decreased DAI score (Fig. 2b, c). While IFN-γ-primed-MSC exosomes infusion showed better efficacy to restore the mice body weight loss and decrease DAI score compared with control ones infusion. The length of colon was measured when the samples were harvested and the results showed that the length of colon significantly decreased in colitis mice, while exosomes infusion significantly decreased colon shortening and IFN-γ-primed-MSC exosomes showed higher efficacy than control exosomes infusion (Fig. 2d). Histologically, the intestines were impaired with inflammatory cell infiltration, aberrant crypt architecture, and epithelial ulceration in colitis mice. Systemic infusion of both control and IFN-γ-primed-MSC exosomes partially restored the impaired intestinal structure, showed as the histological score. Moreover, IFN-γ-primed-MSC exosomes showed superior effects to restore the intestinal structure compared with the control ones (Fig. 2e, f). Furthermore, increased ratio of Th17 cells and decreased ratio of Treg cells were observed in colitis mice (Fig. 2g, h). Infusion of exosomes could block the Th17 cell increase and Treg cell decrease. IFN-γ-primed-MSC exosomes infusion group demonstrated lower Th17 cell ratio and higher Treg cell ratio compared with control exosomes group (Fig. 2g, h). The level of inflammation cytokines TNF-α, IFN-γ, and IL-6 of the colon tissue and lipocalin-2 (LCN2) of feces and serum were significantly elevated in colitis mice. The elevated cytokines in colitis mice were decreased by exosomes infusion, while IFN-γ-primed-MSC exosomes infusion showed higher efficacy than control exosomes (Fig. S2a–e). Taken together, these results indicated that exosomes derived from MSCs showed therapeutic effects for experimental colitis in mice, and the therapeutic effects of exosomes could be enhanced by IFN-γ pretreatment.

### Exosomes regulated Th17/Treg differentiation

**Fig. 3:**
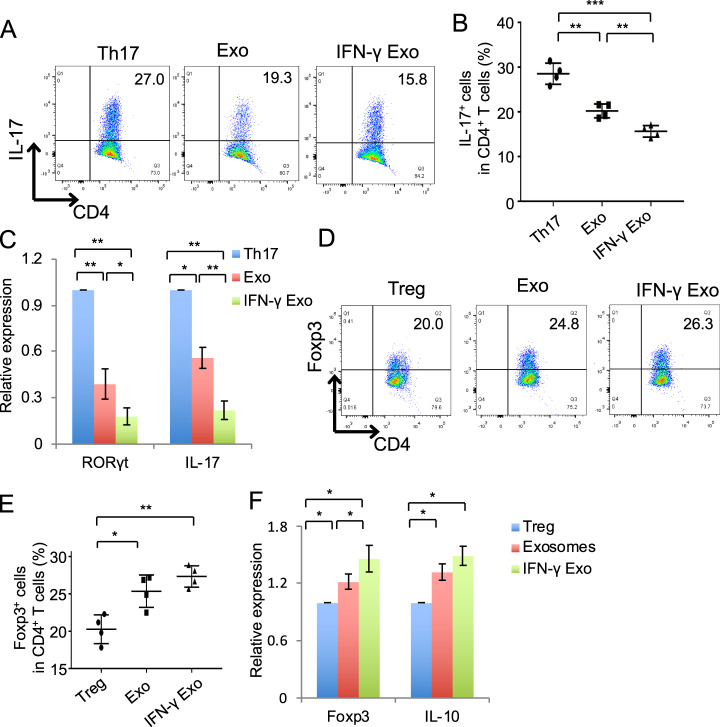
Exosomes treatment inhibited Th17 cell differentiation. **a**, **b** Th17 cell differentiation in vitro after treated by 50 ng/ml control or IFN-γ-primed-MSC exosomes for 3–4 days, assessed by flow cytometry. **c** RORγt and IL-17 expression in Th17 cells, analyzed by qPCR. **d**, **e** Treg cell differentiation in vitro after treated by 50 ng/ml control or IFN-γ-primed-MSC exosomes for 3–4 days, assessed by flow cytometry. **f** Foxp3 and IL-10 expression in Treg cells, analyzed by qPCR. *P < 0.05, **P < 0.01, ***P < 0.001 Exo exosomes; IFN-γ Exo exosomes derived from IFN-γ-primed MSCs.

To explore the underlying mechanism for the immunomodulation of exosomes, naive T cells were isolated and induced under Th17 cell and Treg cell polarization condition in vitro. Either control or IFN-γ-primed MSCs exosomes (50 ng/ml) were added during Th17 or Treg cell differentiation, the results showed that exosomes treatment inhibited Th17 cell differentiation significantly (Fig. 3a, b), accompanied by decreasing the expression of RORγt and IL-17, as assessed by qPCR (Fig. 3c). Moreover, IFN-γ-primed-MSC exosomes showed superior capability to inhibit Th17 cell differentiation (Fig. 3a–c). The level of IL-17 in culture supernatant was also decreased after exosomes treatment. Moreover, the level of IL-17 in IFN-γ-primed-MSC exosomes group was significantly lower than in exosomes treatment group, as assessed by enzyme-linked immunosorbent assay (ELISA) (Fig. S3a). When the exosomes were added to Treg cells, exosomes treatment could promote Treg cells differentiation, and IFN-γ pretreatment enhanced the capability of exosomes to promote Treg cell differentiation (Figs. 3d–f and S3b). T cells apoptosis were not altered by exosomes treatment (Fig. S3c).

### MiR-125a and miR-125b decreased Th17 cell differentiation

**Fig. 4:**
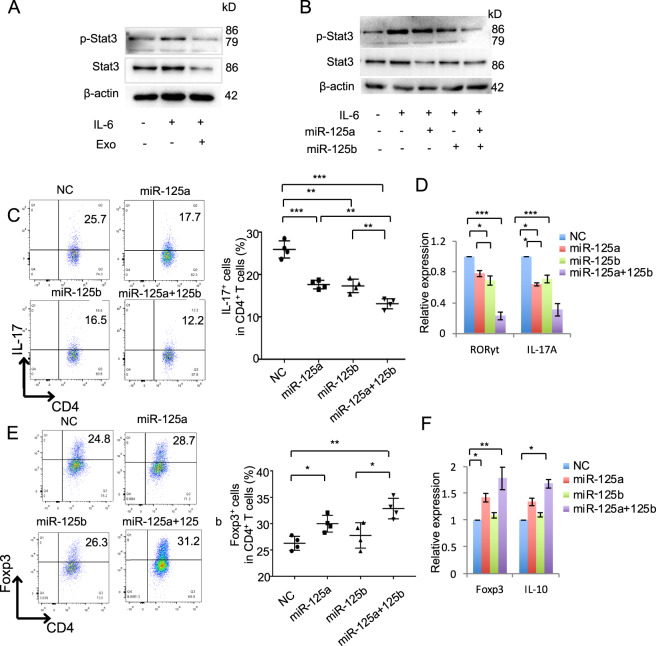
MiR-125a and miR-125b inhibited Th17 cell differentiation. **a** The expression of p-Stat3 and Stat3 in T cells after IL-6 stimulation with or without 50 ng/ml exosomes treatment, as assessed by western blot. **b** The expression of p-Stat3 and Stat3 in T cells with the treatment as indicated. **c** Th17 cell differentiation treated by miR-125a (20 nmol), miR-125b (20 nmol), or miR-125a and miR-125b combination mimics for 3–4 days, analyzed by flow cytometry. **d** The expression of RORγt and IL-17 in Th17 cells in control, miR-125a, miR-125b, or miR-125a and miR-125b combination mimics treatment groups, analyzed by qPCR. **e** Treg cell differentiation treated by miR-125a (20 nM), miR-125b (20 nM), or miR-125a and miR-125b combination mimics, as assessed by flow cytomotry. **f** The expression of Foxp3 and IL-10 in in control, miR-125a, miR-125b, or miR-125a and miR-125b combination mimics treatment groups, as assessed by qPCR. Exo exosomes, NC negative control miRNA. *P < 0.05, **P < 0.01, ***P < 0.001.

As exosomes showed significant inhibition effects on Th17 cells differentiation, which requires the presence of IL-6 and its signal transduction factor Stat3^22^. During Th17 cell differentiation, we treated Th17 cells with exosomes and found that phosphorylation of Stat3 activated by IL-6 and total Stat3 were inhibited by exosomes treatment, as assessed by western blot (Fig. 4a). The therapeutic effects of exosomes are mainly mediated by transferring various miRNAs or growth factors to recipient cells. We using Targetscan and miRDB to predict the miRNA targeting on Stat3, the results showed that twelve putative miRNA was selected by both the Targetscan and miRDB (binding score > 70) database (Fig. S4a and Table S1). Then, we compared the expression of these twelve miRNA in control and IFN-γ-primed MSCs and the results showed that miR-125a (miR-125a-5p) and miR-125b (miR-125b-5p) were significantly elevated in IFN-γ-primed MSCs compared with the control ones (Fig. S3b). To analyze whether miR-125a and miR-125b regulate Th17 cells differentiation, we analyzed the effects of these miRNA on expression of Stat3 and the western blot results showed that miR-125a mimics or miR-125b mimics or their combination treatment could significantly decrease the expression of p-Stat3 and total Stat3 (Fig. 4b). To verify the effects of miR-125a and miR-125b on T-cell differentiation, we transfected miR-125a or miR-125b or their combination mimics to treat T cells during the Th17 and Treg cell differentiation, respectively. The results showed that both miR-125a and miR-125b mimics treatment could inhibit Th17 cell differentiation, and miR-125a and miR-125b combination mimics could enhance their inhibition efficacy (Fig. 4c). MiR-125a and miR-125b mimics treatment inhibited the expression of RORγt in Th17 cells (Fig. 4d) and decreased the level of IL-17 in culture supernatant (Fig. S4c). MiR-125a and miR-125b combination mimics treatment significantly increased Treg cell differentiation (Fig. 4e). MiR-125a or miR-125a and miR-125b combination mimics treatment also increased Foxp3 expression in Treg cells and increased the level of IL-10 in culture supernatant (Figs. 4f and S4d). These data showed that Th17 cell differentiation was inhibited by miR-125a and miR-125b, which represent of important mediator that exosomes repressed Th17 cell differentiation.

### MiR-125a and miR-125b targeted on Stat3

**Fig. 5:**
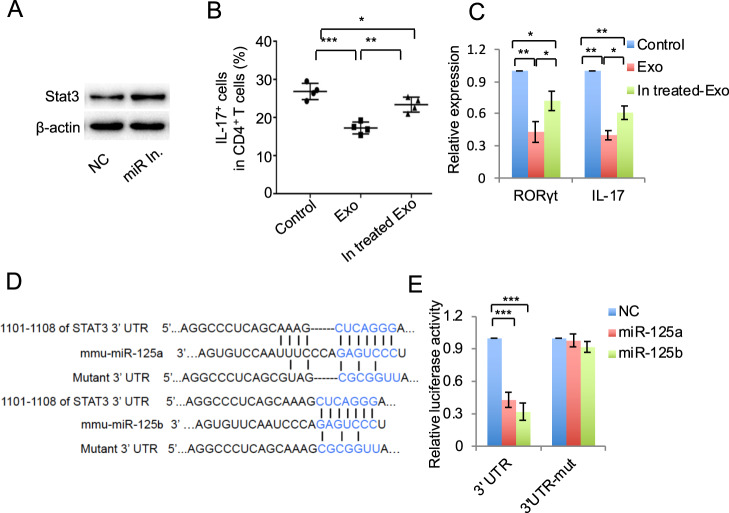
MiR-125a and miR-125b targeted on Stat3. **a** The expression of Stat3 in MSCs with or without miR-125a (20 nmol) and miR-125b (20 nmol) inhibitor treatment, analyzed by western blot. **b, c** Th17 cell differentiation (**b**) and the expression of IL-17 and RORγt (**c**) after control MSCs exosomes or miR-125a and miR-125b inhibitor pretreated MSC exosomes. **d** Sequence of miR-125a and miR-125b and the putative target sites in the 3′-UTR of Stat3 mRNA. Mutation was designed in the complementary binding sites of miR-125a and miR-125b. **e** Luciferase activity analysis. 293T cells were cotransfected with Renilla luciferase control vector and firefly luciferase reporter vector containing either WT or mutant 3′-UTR of Stat3, with or without treatment of miR-125a (20 nM) and miR-125b (20 nM) mimics for 24 h. Exo exosomes, In. treated Exo exosomes derived from miR-125a and miR-125b inhibitor pretreated MSCs. *P < 0.05, **P < 0.01, ***P < 0.001.

To further verify the effects of miR-125a and miR-125b on Th17 cell differentiation, we used miR-125a and miR-125b inhibitor to pretreat MSCs and then to isolate exosomes. These miR-125a and miR-125b inhibitor pretreated exosomes were used to treat Th17 cells, and the results showed that the efficacy to inhibit Th17 cell differentiation by exosomes was partially decreased by miR-125a and miR-125b inhibitor pretreatment (Figs. 5a–c and S5). To test whether miR-125a and miR-125b direct target on Stat3, we constructed the luciferase report vectors that contain the putative normal or mutant miR-125a or miR-125b binding sites within 3′-UTR, respectively (Fig. 5d). The results showed a significant reduction in luciferase activity by cotransfection of luciferase report vectors containing normal binding sites and miR-125a or miR-125b mimics, compared with the vector containing mutant 3′-UTR cotransfection groups (Fig. 5e). These results verify that miR-125a and miR-125b target on Stat3 to repress Th17 cell differentiation.

### MiR-125a and miR-125b agomir infusion attenuated colitis in mice

**Fig. 6:**
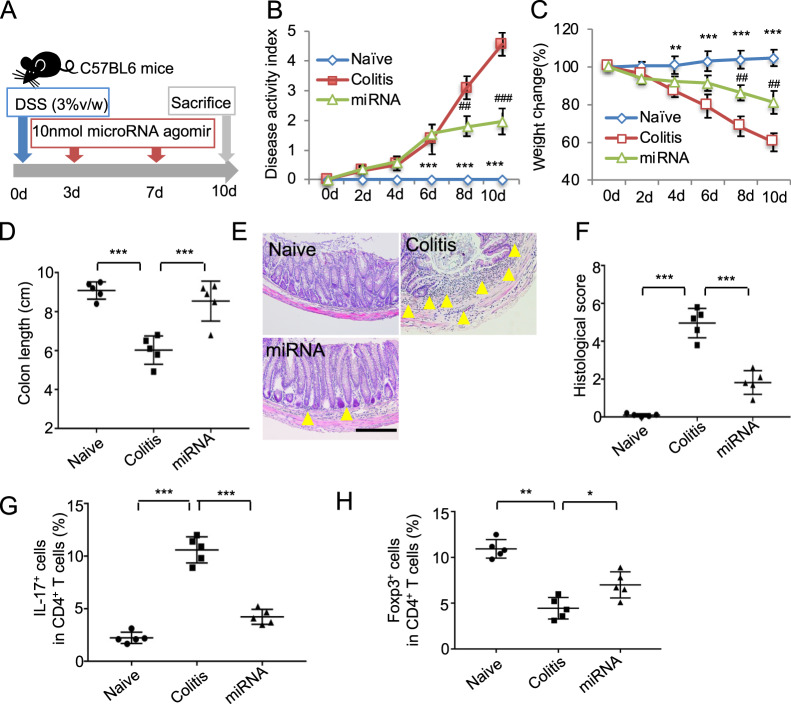
MiR-125a and miR-125b agomir infusion attenuated colitis in mice. **a** The schema of miRNA agomir treat for colitis (*n* = 5). **b–h** The disease activity score change (**b**), mice body weight change (**c**), the length of colon (**d**), the histological staining of colons (**e**), the histological score (**f**), Th17 cells ratio (**g**), and Treg cell ratio (**h**) in control, colitis, and miR-125a and miR-125b (miRNA) agomir treatment group. Scale bar: 100 μm (*n* = 5), d days, DSS dextran sulfide sodium. The yellow triangle: inflammatory cells infiltration. *P < 0.05, **P < 0.01, ***P < 0.001. ^#^miRNA vs colitis; *naive vs colitis.

**Fig. 7:**
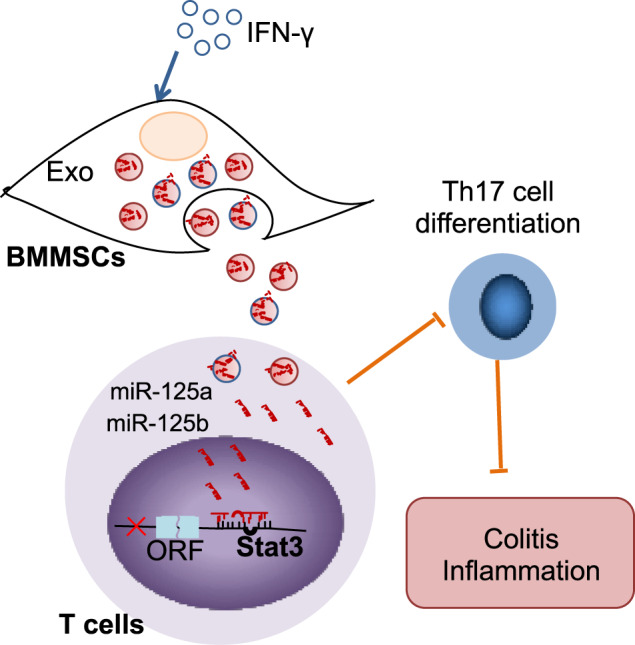
The schema of exosomes derived MSCs to attenuate colitis. Mice colitis could be ameliorated by MSC exosomes infusion, which transferred the miR-125a and miR-125b to T cells targeting on Stat3 to inhibit Th17 cell differentiation. IFN-γ pretreatment to MSCs promoted the levels of miR-125a and miR-125b in exosomes to elevate their therapeutic effects.

To confirm the effects of miR-125a and miR-125b on inhibiting Th17 cells, the miR-125a and miR-125b agomir were infused to experimental colitis in mice (Fig. 6a). Consistent with exosomes treatment, miR-125a and miR-125b agomir injection could restore the body weight loss of colitis mice, the decreased DAI score, and colon shortening (Fig. 6b–d). Infusion of miR-125a and miR-125b agomir also restored the impaired intestinal structure and the histologic score (Fig. 6e, f). Moreover, miR-125a and miR-125b agomir injection decreased the Th17 cell ratio in colitis mice (Fig. 6g). Treg cell ratio in miR-125a and miR-125b agomir injection group was slightly increased (Fig. 6h). The elevated level of inflammation cytokines IL-6, TNF-α, IFN-γ in colon, and LCN2 in feces and serum of colitis mice were decreased by miR-125a and miR-125b agomir injection (Fig. S6a–e). These experiments verified the therapeutic effect of miR-125a and miR-125b, which repressed Th17 cell differentiation *via* targeting Stat3 and shed light on the novel mechanism for exosomes therapy to inflammation diseases (Fig. 7).

## Discussion

IBD, a chronic, relapsing, and tissue-destructive colon disorder, has harmed millions of people’s health worldwide^23^. Regular therapies for IBD usually lead to serious side effects, highlighting the unmet need for alternative strategy to treat IBD. Recently, different biological therapies have been reported and demonstrated superior efficacy and safety^24^. It has been reported that MSCs derived from different tissues such as bone marrow MSCs^25^, MSC from periodontal ligament^26^, and MSC from adipose tissue^27^ could alleviate colitis. However, some researcher raised the concerns about the fate of stem cells infused to body. Exosomes derived from MSCs, providing tissue repair and immune regulatory properties as MSCs, have less cytotoxicity and bio-hazardous potential, and are also not easily degraded^28^. In present study, we showed that exosomes derived from mouse BMMSCs showed therapeutic effects for experimental colitis, which are consistent with the previous report using human BMMSCs^14^. As the therapeutic effect of exosomes was dose dependent, it is an urgent medical need to increase the number or property of exosomes. Several researches have investigated the use of different stimulus such as cytokine, hypoxia, gene modification to improve the production, and function of exosomes^29–31^. Our results showed that exosomes derived from IFN-γ-primed MSCs showed higher efficacy to treat colitis compared with control exosomes, with decreased Th17 cell, and promoted Treg cell differentiation. IFN-γ treatment upregulated the expression of CD81 and CD9 in exosomes. These data indicate that IFN-γ prime may be an alternative to increase therapeutic effects of exosomes, which also shed light the strategy to enhance the properties of exosomes. Further investigation was needed to explore the more effective strategy to promote the properties of exosomes.

The pathogenesis of IBD is highly associated with imbalance of adaptive immunity. The IL-17 producing subset of CD4^+^ T effector cells, termed “Th17” cells, is strongly implicated in the pathogenesis of IBD^32^. This connection has led to IBD therapies targeting on the pathways essential for Th17 differentiation, such as anti-IL-23, anti-IL-23R, and pro-IL-22 agents^33^. Our results showed that the ratio of Th17 cells in the spleens of colitis mice was decreased after treatment by exosomes in vivo. Exosomes could decrease Th17 differentiation *via* inhibiting Stat3 in vitro. These results indicate that the therapeutic effects of exosomes were partly mediated by the downregulation of Th17 cells. The ratio of Treg cells was increased after exosomes infusion in colitis mice. As the reciprocal regulation of Th17 and Treg cells^34^, whether this increase of Treg cells is direct or the subsequent result to Th17 cell alteration need to be further illustrated. It is also reported that MSC-derived exosomes showed therapeutic effect for Th17 cell dominant EAE model in mice^13^. These results shed light that exosomes may be one of the promising alternative targeting on Th17 cell-related immune diseases.

Exosomes has showed promising therapeutic effects on variety disease due to its biological functions in immune response, anti-inflammation, and anti-infection^35,36^. The mechanism for the biological function of exosomes should be context dependent and elusive. Here, we showed that miR-125a and miR-125b in exosomes derived from MSCs targeted on Stat3 to inhibit Th17 differentiation, then resulted in alleviating the symptoms of colitis in mice. Moreover, IFN-γ prime upregulated the expression of miR-125a and miR-125b in MSCs to enhance the therapeutic effects of exosomes. These results were consistent with the previous study that miR-125a^−/−^ mice developed more severe colitis induced by 2,4,6-trinitrobenzenesulfonic acid (TNBS) compared with WT mice^37^. The role of miRNA on IBD patient diagnosis and therapy on IBD experimental animal models has been attracted more attention recently. For instance, IBD patients showed higher level of miR-16, miR-21, and miR-223 and miR-155 in feces compared with controls, which correlate with disease activity^38^. AntagomiR-148a-mediated reduction of Th1 cells selectively ameliorated chronic colitis without affecting the protective immunological memory^39^. Extracellular vesicles containing miR-146a ameliorates experimental TNBS caused colitis by targeting TRAF6 and IRAK139. MiR-106a knockout attenuated chronic murine ileitis *via* promoting Treg induction and suppressive function and IL-10 production^40^. These studies indicated that various miRNAs played critical role in the pathogenesis and treatment of IBD, and their effects may vary based on the age (children or adults), the symptoms (chronic or acute), the status of IBD (active or inactive), and the pathogenesis of colitis and so on^41^. For the mechanism how miRNA take participated in the IBD, we found that miR-125a and miR-125b inhibit Th17 cells by targeting on Stat3. Ge et al.^37^ showed that miR-125a inhibit human Th1 and Th17 cell differentiation by targeting on EST-1. Moreover, a variety of studies have shown that a number of miRNAs, such as miR-27a^42^, miR-106a^43^, miR-10a^44^, and miR-210^45^ possess inhibitory effects on differentiation of Th17 cells. T-cell apoptosis targeted by miRNA may be involved in the pathogenesis of colitis, such as miR‐665 enhanced apoptosis and exacerbates colitis in IBD by inhibiting XBP1^46^. The precise role of miRNAs in IBD needs further investigations, as well as the diagnostic, the underlying mechanisms, and therapeutic modalities to IBD.

Because characteristics of exosome depending on the status of MSCs they derived from. It has been reported that exosomes derived from proinflammatory cytokines pretreated-MSCs showed different proteins, miRNA, and cytokine profile such as IFN-γ, TNF-α, PGE2, IDO, miR-34a, and miR-146a^29,47,48^. Here, we showed that IFN-γ pretreatment increased the levels of miR-125a and miR-125b in exosomes from MSCs to increase the therapeutic capacity. As far as we know, this is the first time to illustrate that the therapeutic effects by applying exosomes pretreated with IFN-γ to colitis model, which mediated by increasing the level of miR-125a and miR-125b. Whether other proteins or miRNAs were altered in exosomes derived from different primed MSCs and their effects need to be further studied.

Summarily, our study shows that exosomes derived from MSCs attenuate DSS-induced colitis in mice. IFN-γ-primed-MSC exosomes exert superior therapeutic efficacy for colitis, which are mainly mediated by increasing the expression of miR-125a and miR-125b. Mechanically, miR-125a and miR-125b in exosomes inhibited Th17 cells differentiation by target on Stat3. Briefly, our findings provide insight into promote the immune regulation of MSC exosomes and suggest a promising approach to Th17 cell aberrant related diseases treatment.

### Experimental procedures

#### Mice and antibody

Female C57BL/6J mice were purchased from River Laboratory (Beijing Vital River Laboratory Animal Technology, China). All the animal experimental protocols were approved by the Institutional Animal Care and Use Committee at Peking University (LA2019-77)

The antibodies to anti-p-Stat3 (9145s) and anti-Stat3 (12640s) were purchased from cell signaling. Anti-β-Actin antibody was purchased from Sigma-Aldrich. Anti-CD63 (sc-5275), anti-CD9 (sc-9148), and anti-CD81 (sc-9158) antibodies were purchased from Santa Cruz Biotechnology. Anti-mouse CD4-PerCP (100538) and IL-17A-PE (506904) were purchased from Biolegend (San Diego, CA). Anti-Foxp3-PE (12-4771-82), Anti-CD4-APC (17-0041-82), anti-IL-17A-APC (17-7179-42), anti-Foxp3-PercP (45-5773-82), anti-CD105-PE (12-1051-82), CD45-PE (25-0454-82), CD73-PE (12-0739-42), CD34-PE (12-0349-42), and CD90-PE (48-0902-82) antibodies were purchased from eBioscience (San Diego, CA, USA). Purified anti-mouse CD3ε (553057) and CD28 (553295) antibodies were purchased from BD Bioscience (Franklin Lakes, NJ). MiRNA inhibitors or mimics and agomir (miR-125a mimics (miR10000135-1-5); miR-125b mimics (miR10000136-1-5); miR-125a inhibitor (miR20000135-1-5); miR-125b inhibitor (miR20000136-1-5); miR-125a agomir (miR414619171151-4-5); miR-125b agomir (miR4161128021318-4-5); micrON mimic negative control (NC) (miR1N0000001-1-5); micrOFF inhibitor NC (miR2N0000001-1-5); and micrON agomir NC (miR04102-4-5)) were purchased from RiboBio (Guangzhou, China).

### Isolation and culture of mouse MSCs

Mouse femurs and tibias were collected and flushed out the bone marrow cells with 2% fetal bovine serum (FBS) in PBS. The nuclear cells (ANC) were seeded (1 × 10^7^ cells per dish) in 100 mm culture dishes (Corning,) and incubated at 37 °C under 5% CO_2_. After 2 days culture, the floating cells were washed by PBS and adherent cells were cultured in complete alpha minimum essential medium as previously reported^49^. After around 80–90% confluent, the cells were passaged for further experiment.

### Isolation of exosomes

MSCs were cultured in complete medium with FBS (depleted exosomes by 2 h centrifugation at 100,000 × *g*) for 2 days. Exosome-depleted culture supernatant of BMMSCs was collected. Differential centrifugation were used to extract exosomes from culture supernatants of 1.2 × 10^7^ BMMSCs, as previously described^49,50^, briefly centrifuged at 300 × *g* for 10 min, 3000 × *g* for 10 min, 20,000 × *g* for 30 min, and 120,000 × *g* for 70 min to collect the exosomes. To quantitate the number of exosomes, EXOCEP exosome quantitation kit (System Biosciences Inc.) was used following the manufacturer’s instruction

### Transmission electron microscopy

For analyzing BMMSCs by TEM, BMMSCs were collected and treated as per our previous report^49^. The samples were photographed with an FEI Tecnai spirit transmission electron microscope (FEI Tecnai Spirit 120kv).

For exosomes analysis by TEM, the isolated exosomes derived from 1 × 10^6^ MSCs were suspended in 2.5% glutaraldehyde and loaded onto copper grids. The grids were examined using the FEI Tecnai spirit TEM (FEI Tecnai Spirit 120kv).

### Treg and Th17 cells differentiation in vitro

Naive T cells were collected by sorting CD4^+^CD25^−^CD62L^hi^CD44^low^ cells from mouse spleens and stimulated with anti-CD3 (5 μg/ml) and anti-CD28 (5 μg/ml) with different cytokine cocktails. The T cells were cultured 3–4 days under hTGF-β1 (R&D Systems; 2 ng/ml) with IL-2 (PeproTech; 10 U/ml) for Treg cell differentiation and hTGF-β1 (0.5 ng/ml) with IL-6 (PeproTech; 20 ng/ml) for Th17 differentiation as previously reported^4^. For exosomes treatment, 50 ng/ml exosomes derived from MSCs were added in the culture medium during the T-cell induction. For microRNA treatment, the final concentration of 20 nM miR-125a, miR-125b mimics, or NC mimics were transfected before T-cell differentiation.

### MiRNA mimics and inhibitor transfection

For miR-125a and miR-125b mimics and inhibitor transfection in T cells, the Amaxa Mouse T cell Nucleofector kit was used according to the manufacturer’s instructions (Lonza). Briefly 2 × 10^6^ FACS sorted naive CD4^+^T cells were transfected with 20 nM (final concentration) miR-125a and miR-125b or NC mimics using Lonza 4D Nucleofector. Then, the naive CD4^+^ T cells were stimulated with anti-CD3 and anti-CD28 and the polarization inducing cytokines for Treg and Th17 cells differentiation. For miR-125a and miR-125b inhibitor transfection in MSCs, the 2 × 10^5^ BMMSCs were transfected with miR-125a and miR-125b or NC inhibitor (20 nmol) using riboFECT™ CP Reagent reagent (RiboBio) according to the manufacturer’s instructions and then cultured 48 h for exosomes isolation.

### Western blotting

Total protein from cells or isolated exosomes was lysed using M-PER mammalian protein extraction reagent and was quantified using a protein concentration assay. For analysis of exosomes, exosome protein was isolated from the culture supernatant of 4 × 10^6^ cells, and 10 μg of total exosome proteins was loaded. For western analysis of cells, the 10 μg proteins were loaded for western blot as per our previously described report^26^. The relative density was measured using ImageJ 1.49v software (Wayne Rasband).

### MiRNA prediction

MiRDB database (http://mirdb.org/) and TargetScan (http://www.targetscan.org) were used to predict the putative miRNAs target on Stat3.

The conserved 8-mer and 7-mer sites matched the seed region were choosed from TargetScan and the ones binding score equal or more than 70 were selected from miRDB database.

### Real-time PCR

Total RNA from control MSCs was extracted from the cultured cells using Trizol (Invitrogen). To analyze the expression of mRNA, SuperScript III Reverse Transcriptase (RT) kit (Invitrogen) was used to synthesis cDNA. qPCR was performed using the specific primer (listed in Table S2) as previously described^26^. The level of mRNA expression was normalized to glyceraldehyde 3-phosphate dehydrogenase. For qPCR of miRNA, Bulge-Loop^TM^ miRNA qRT-PCR Starter Kit (RiboBio) was used to synthesize the cDNA. The Bulge-Loop^TM^ miRNA qPCR primer were used and purchased from RiboBio (Guangzhou, China). The small nuclear RNA U6 was used as an endogenous control. SYBR Green was used to perform real-time PCR. A CFX96 Real-Time PCR System (Bio-Rad) was used for qPCR analysis.

### Luciferase assay

The fragment of control and mutant 3′-UTR mRNA sequences of STAT3 containing the miR-125a and miR-125b binding site were amplified by Genewiz in China, and the amplified fragments were cloned in to pMIRGLO vector between the SacI site and Xbal site.

For luciferase assay, 5 × 10^4^ cells per well HEK-293T cells were seeded in a 24-well plate. After 24 h culture, the cells were transfected with Renilla luciferase control vector, luciferase reporter vector, and either miRNA NC or miR-125a mimics or miR-125b mimics (20 nM) for 24 h using riboFECT™ CP Reagent reagent (RiboBio) according to the manufacturer’s instructions. The luciferase assay was performed using the dual luciferase assay kit (Promega) following the manufacturer’s instruction.

### Enzyme-linked immunosorbent assay

Colon tissues from the naive, colitis, Exo, IFN-γ Exo, and miRNA treated mice were collected for the detection of TNF-α, IFN-γ, and IL-6. Culture supernatant of Th17 and Treg cells were collected for the detection of IL-17 and IL-10. TNF-α, IFN-γ, IL-6, and IL-17 ELISA kit were from eBioscience. Fecal samples were collected and reconstituted in PBS containing 0.1% Tween 20 and vortexed for 20 min to get a homogenous fecal suspension in 100 mg/ml as previously described^51^. Then, were centrifuged for 10 min at 12,000 rpm and 4 °C to get the clear supernatants. Serum were collected from mice and diluted for ten times with kit-recommended reagent diluent buffer. LCN2 levels were estimated using Duoset murine Lcn-2 ELISA kit (R&D Systems). All the procedures were performed according to the manufacturer’s instruction.

### DSS-induced mouse colitis

To assess the therapeutic effects of exosomes and miRNA, 3% (w/v) DSS (MP Biochemicals) was dissolved into drinking water to fed 8–10 weeks old C57BL/6J mice for 10 days to induce acute colitis according to the previously described reports^25,52^. For exosomes treatment, 200 μg of exosomes was i.v. injected into mice 3 days after DSS administration (*n* = 5). For miRNA agomir injection, 10 nmol miR-125a and miR-125b or miRNA agomir NC were injected i.v. on day 3 and day 7, respectively. DAI was scored (0–5) by evaluating the mice weight loss, stool consistency/diarrhea, and presence of fecal bleeding^21,53^. At day 10, mice were sacrificed by CO_2_ euthanasia, and the colons were fixed with 4% paraformaldehyde and embedded sections were prepared for H&E staining. Histological scores were blindly determined as previously described by evaluating the tissue damage (score 0–3) and inflammatory cell infiltration (score 0–3) in colitis mice^21,54^.

### Statistics

All results are presented as the mean and standard deviation (mean ± S.D.) Comparisons between two groups were analyzed using independent unpaired two-tailed Student’s *t* tests, and comparisons between more than two groups were analyzed using one-way ANOVA by SPSS 19.0 software. Exact number of mice used in each group was indicated in the figure legends. Data were assessed for normal distribution and similar variance between groups prior to further statistical analysis. *P* values less than 0.05 were considered statistically significant.

## Supplementary information

Supplementary Figure legend

Supplementary Figure. S1

Supplementary Figure. S2

Supplementary Figure. S3

Supplementary Figure. S4

Supplementary Figure. S5

Supplementary Figure. S6

Supplementary Figure. S7

Supplementary Figure. S8
