# Supplementary material for: IFN-γ promoted exosomes from mesenchymal stem cells to attenuate colitis via miR-125a and miR-125b
Source: Cell Death Dis. 2020 Jul 30;11(7):603. doi: 10.1038/s41419-020-02788-0 (PMC7393506; doi:10.1038/s41419-020-02788-0)
Supplement: Supplementary file 1 — Supplementary Figure legend [file 41419_2020_2788_MOESM1_ESM.docx]

Supplementary Figure legend

Figure. S1. The expression of mesenchymal stem cells markers CD105, CD90 and CD73, and hematopoietic stem cells marker CD45 in control and IFN-γ primed MSCs. (Related to Fig. 1)

Figure. S2 IFN-γ primes could promote exosomes to attenuate colitis in mice. (**A-E**)The level of TNF-α (**A**), IFN-γ(**B**), IL-6 (**C**)of colon and lipocalin-2 (LCN-2) of feces (**D**) and serum(**E**) in naïve, DSS，and control MSC exosomes and IFN-γ-primed MSC exosomes infusion groups. *P < 0.05, **P < 0.01, ***P < 0.001. (Related to Fig. 2)

Figure. S3 Exosomes treatment inhibited Th17 cell differentiation. (A) The level of IL-17 in culture supernatant of control, control MSC exosomes and IFN-γ-primed MSC exosomes treated Th17 cells, as assessed by ELISA. (B) The level of IL-10 in culture supernatant of control, control MSC exosomes and IFN-γ-primed MSC exosomes treated Treg cells, as assessed by ELISA. (C) T cell apoptosis after control MSC exosomes or IFN-γ-primed MSC exosomes treatment. *P < 0.05, **P < 0.01, ***P < 0.001. (Related to Fig. 3)

Figure. S4 Putative miRNA targeted on Stat3. (A) The data base of Targetscan and miRDB were used to scan the miRNA targeted on Stat3. There were 12 putative miRNA were selected by both Targetscan and miRDB (binding score>70). (B) The expression of the 12 selected putative miRNA in control and IFN-γ primed-MSCs, as assessed by qPCR. (C, D) The level of IL-17 and IL-10 in culture supernatant of NC, miR-125a, miR-125b and miR-125a and miR-125b combiniation mimics treated Th17 and Treg cells, respectively. *P < 0.05, **P < 0.01, ***P < 0.001. (Related to Fig 4)

Figure. S5 The level of IL-17 in culture supernatant of control, Exo and In. treated Exo induced Th17 cells, as asessed by ELISA. *P < 0.05, **P < 0.01, ***P < 0.001. Exo: Exosomes; In. treated Exo: Exosomes derived from miR-125a and miR-125b inhibitor pre-treated MSCs. (Related to Fig 5)

Figure. S6 MiR-125a and miR-125b agomir infusion attenuated colitis in mice. (**A-E**)The level of TNF-α (**A**), IFN-γ(**B**), IL-6 (**C**) of colon and lipocalin-2 (LCN-2) of feces (**D**) and serum(**E**) in naive, DSS，and miRNA treated mice. *P < 0.05, **P < 0.01, ***P < 0.001. miRNA: miR-125a and miR-125b agomir. (Related to Fig. 6)
