# Supplementary figures and images for: IFN-γ promoted exosomes from mesenchymal stem cells to attenuate colitis via miR-125a and miR-125b
Source: Cell Death Dis. 2020 Jul 30;11(7):603. doi: 10.1038/s41419-020-02788-0 (PMC7393506; doi:10.1038/s41419-020-02788-0)

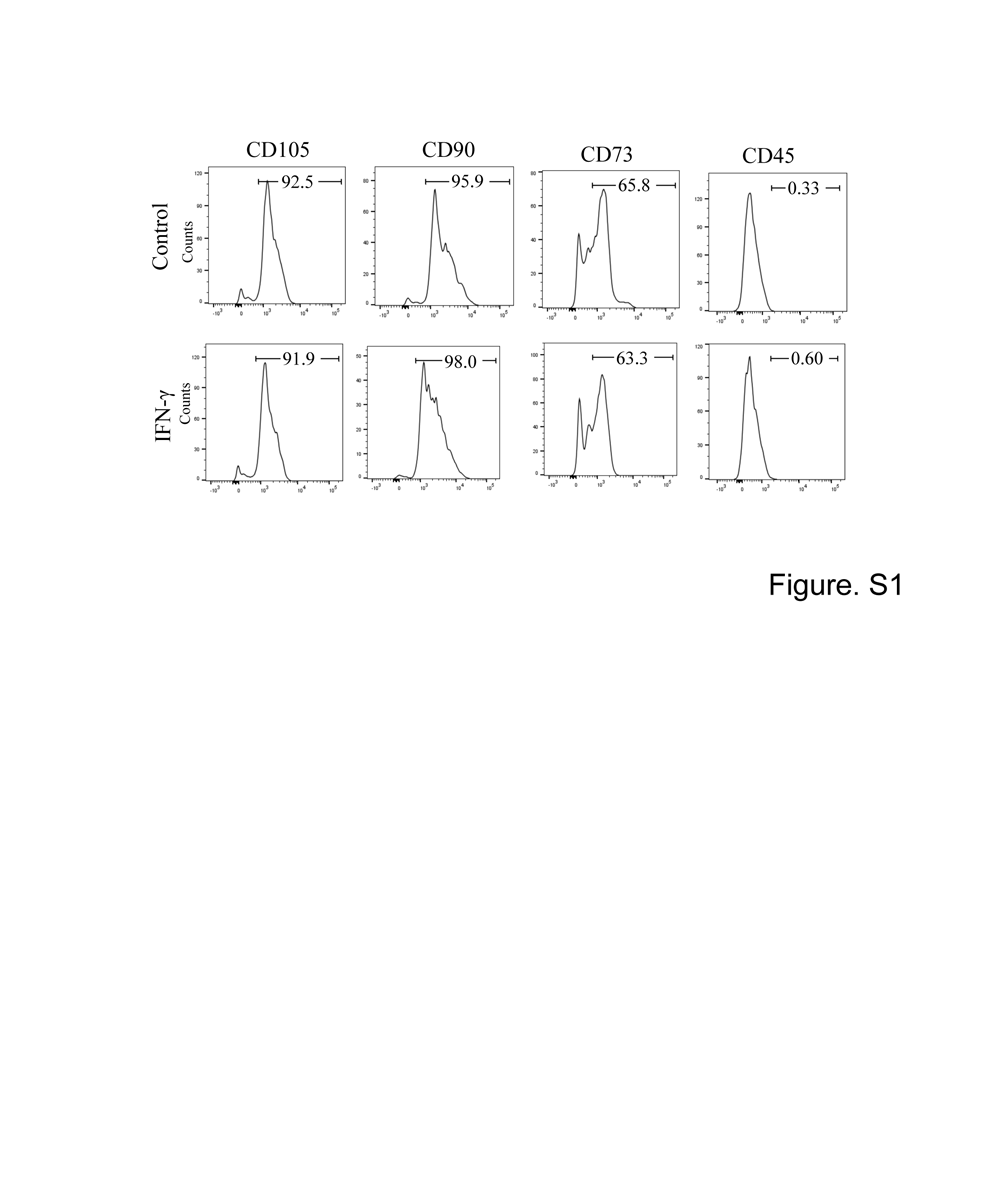

Supplement: Supplementary file 2 — Supplementary Figure. S1 [file 41419_2020_2788_MOESM2_ESM.tif]

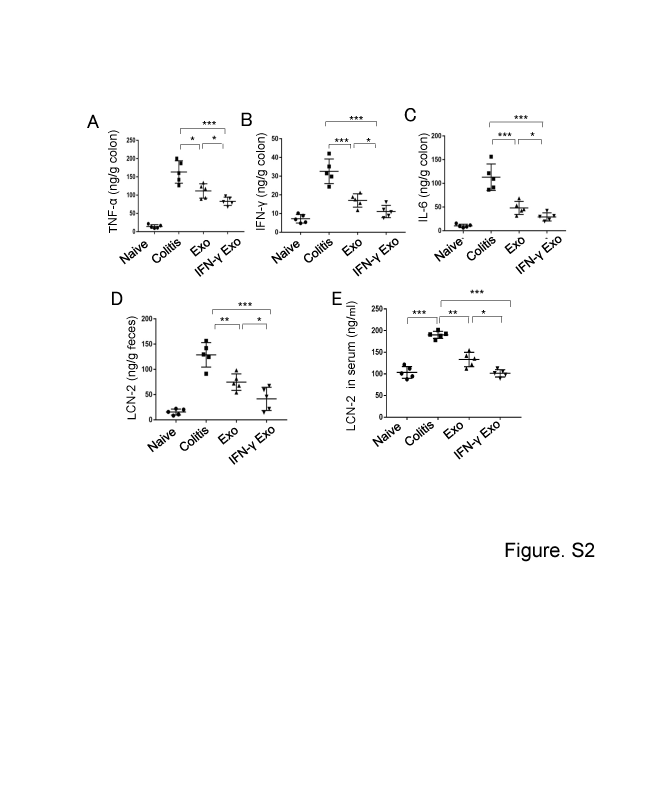

Supplement: Supplementary file 3 — Supplementary Figure. S2 [file 41419_2020_2788_MOESM3_ESM.tif]

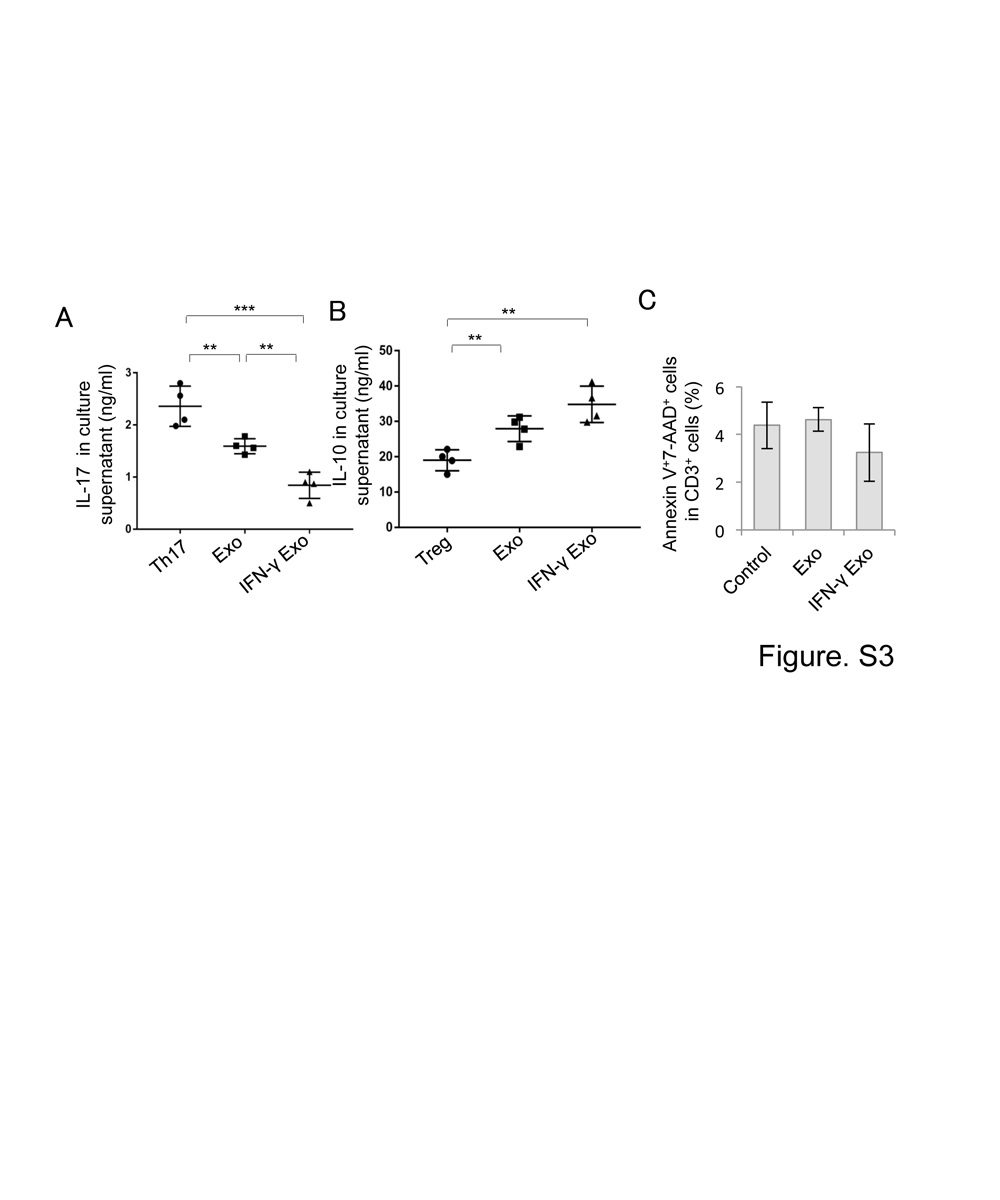

Supplement: Supplementary file 4 — Supplementary Figure. S3 [file 41419_2020_2788_MOESM4_ESM.tif]

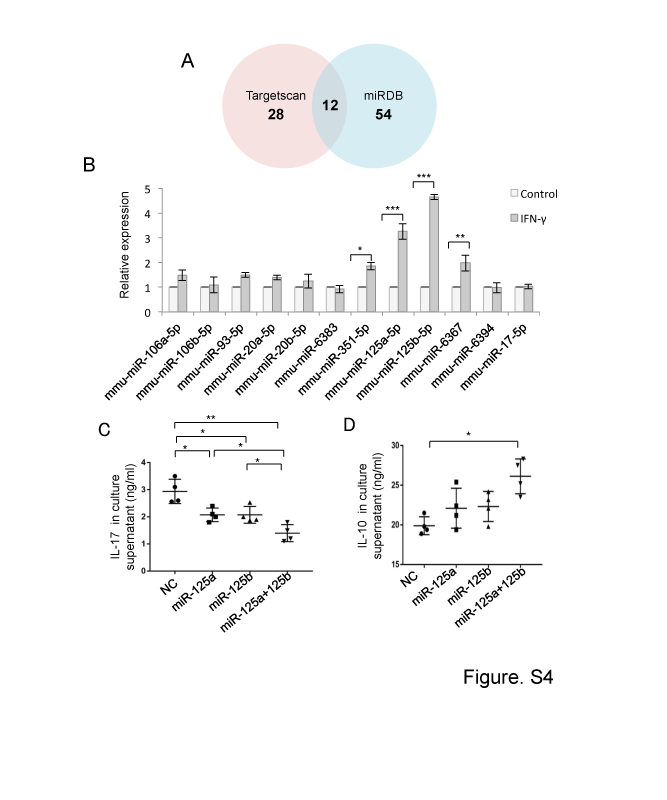

Supplement: Supplementary file 5 — Supplementary Figure. S4 [file 41419_2020_2788_MOESM5_ESM.tif]

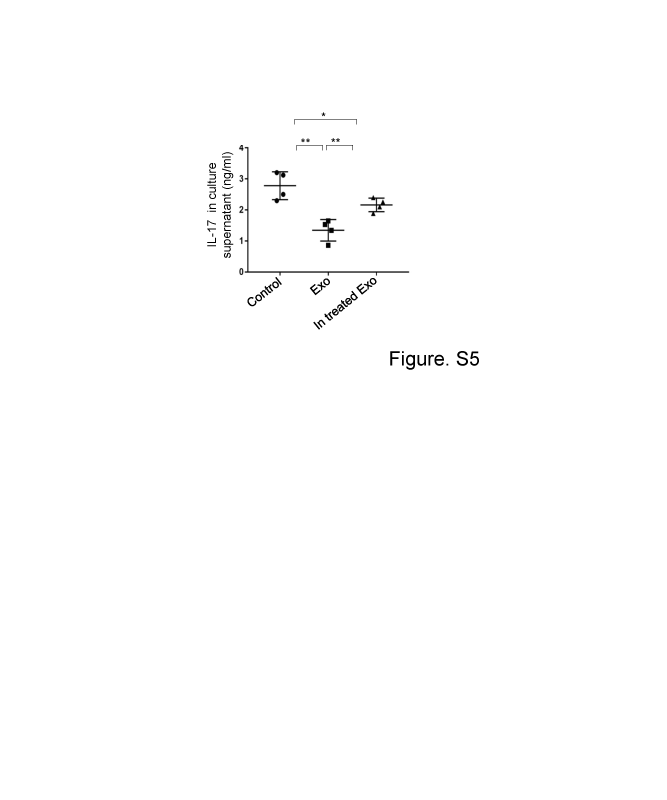

Supplement: Supplementary file 6 — Supplementary Figure. S5 [file 41419_2020_2788_MOESM6_ESM.tif]

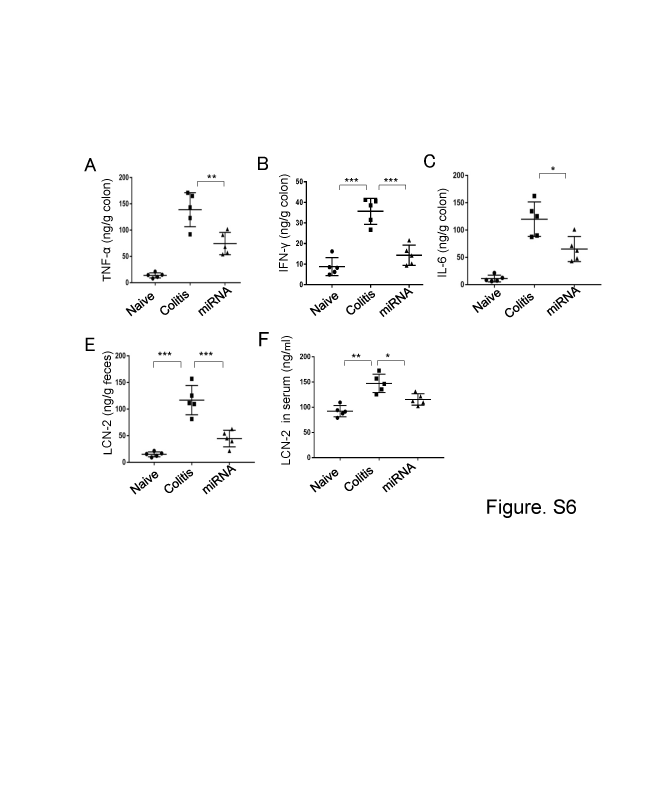

Supplement: Supplementary file 7 — Supplementary Figure. S6 [file 41419_2020_2788_MOESM7_ESM.tif]

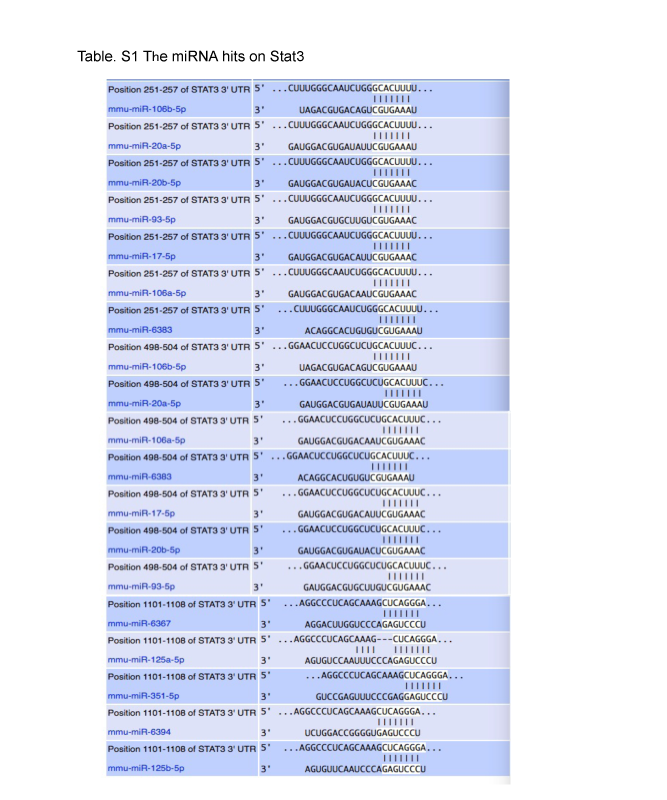

Supplement: Supplementary file 8 — Supplementary Figure. S7 [file 41419_2020_2788_MOESM8_ESM.tif]

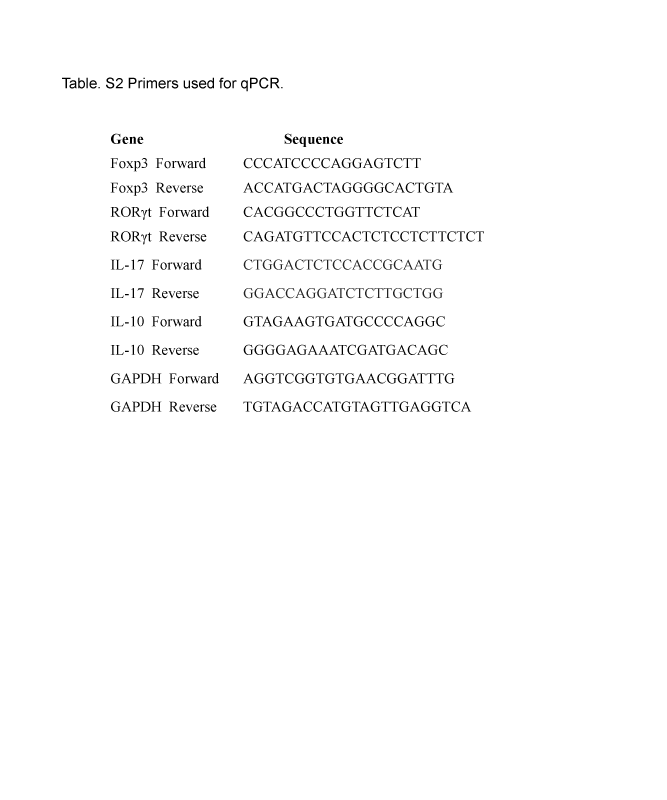

Supplement: Supplementary file 9 — Supplementary Figure. S8 [file 41419_2020_2788_MOESM9_ESM.tif]
